# Innovations for an Aging Society through the Lens of Patent Data

**DOI:** 10.1093/geront/gnad015

**Published:** 2023-07-27

**Authors:** Reuben Ng, Nicole Indran

**Affiliations:** Lee Kuan Yew School of Public Policy, National University of Singapore, Singapore, Singapore; Lloyd’s Register Foundation Institute for the Public Understanding of Risk, National University of Singapore, Singapore, Singapore; Lee Kuan Yew School of Public Policy, National University of Singapore, Singapore, Singapore

**Keywords:** Age stereotypes, Anti-aging, Healthy aging, Inventions, Patents

## Abstract

**Background and Objectives:**

An aging population creates fertile ground for devising innovations for older adults. By using patents as a proxy for inventive activity, this study sets the stage for understanding the latest innovations being designed for the older population. Insights will pave the way for a better understanding of inventions that could render society more age-friendly on the innovation front.

**Research Design and Methods:**

To identify the latest innovations targeted at the older population, we collected all patents (*N* = 326) issued in 2021, specifically those issued between January 5th and December 28th. Upon removing irrelevant data, 120 patents were retained in the data set. Both inductive and deductive modes of reasoning informed our content analysis of the data.

**Results:**

Three themes surfaced. About half (49.2%) of the patents focused on “Preventive Health, Safety, and Independence” (Theme 1). About 38.3% pertained to “Anti-Aging” (Theme 2) and 12.5% were about the “Pathologization of Old Age” (Theme 3).

**Discussion and Implications:**

This is the first study that evaluates the state of innovations for an aging population. While there are inventions aimed at optimizing the well-being of older adults, there are also those designed due to beliefs that see old age as a problem to solve. As the world experiences a demographic shift, it is imperative that collective ingenuity be harnessed to build a society conducive to all facets of the aging experience.

About one fifth of the American population is forecast to be aged 65 or older by 2030 (Vespa et al., 2018). That global life spans have essentially doubled over the last century is a crowning achievement in efforts to improve public health. However, epithets such as “silver tsunami,” “gray wave,” and “agequake” metaphorize an aging population as a crisis of epic proportions (Barusch, 2013), consequently detracting from the miracle of such an accomplishment. Whether an aging population is a boon or a bane depends on how society responds and adapts to it. To reap the benefits of an aging population, there is a need to develop inventions that cater to the needs of older adults. In this study, we explore the various innovations that have emerged in relation to the older demographic by analyzing a data set of related patent grants.

This study is conceptually significant in that it is the first to explore a data set of patented publications in the gerontological field. As people live longer, there is an urgent need to improve well-being across the life span. This entails prioritizing the needs of older adults and creating products and services conducive to all aspects—physical, cognitive, social, and psychological—of aging. From a practical perspective, this study sets the stage for understanding the latest innovations being designed for the older population by using patents as a proxy for inventive activity or innovation (Basberg, 1987). Insights will pave the way for a better understanding of inventions that could render society more age-friendly on the innovation front.

Being a relatively affluent demographic, older adults are major players in the economy (Arensberg, 2018). The term “silver economy” was minted precisely to encapsulate the opportunities arising from economic activities that serve the needs and demands of older persons (World Bank, 2020). Statistics indicate that the silver economy is becoming an increasingly powerful force. In 2014, the silver economy in the United States was valued at $7.1 trillion, which places it just behind the economy of both the United States and China if it were considered a country (Nahal & Ma, 2016). Estimates put the value of the silver economy at USD$15 trillion in 2020 (Nahal & Ma, 2016).

As the population ages, governments face a greater obligation to design and transform their health and social delivery systems to meet the needs of older individuals (WHO Centre for Health Development, 2015). This was the impetus for the inaugural Global Forum on Innovation for Aging Populations held by the World Health Organization (WHO) in 2013, during which 170 experts from 21 countries discussed various technological and social innovations to facilitate healthy aging (World Health Organization, 2013).

The concept of “healthy aging” has gathered pace over the decades. A complex construct encompassing multiple dimensions (Abud et al., 2022), healthy aging denotes more than just the absence of disease. Though there is no one way to define the concept (Rodriguez-Laso et al., 2018), it can generally be understood as the process of optimizing opportunities for improving as well as preserving health and well-being (Peel et al., 2005). In 2020, the WHO and United Nations (UN) member states launched the UN Decade of Healthy Ageing (2021–2030), a 10-year global plan of action to ensure that older adults can live long and healthy lives (Amuthavalli Thiyagarajan et al., 2022). The Decade of Healthy Ageing aims to improve the lives of older persons by addressing four areas of action: (i) creating age-friendly environments; (ii) combating ageism; (iii) delivering integrated care services; (iv) providing access to long-term care (World Health Organization, 2020).

Though plans are afoot to accommodate an older population, understanding what these needs are remains underprioritized by the for-profit sector (Coughlin, 2017). Products and services targeted at this segment still face criticism for being outdated (Terrell, 2019). Older adults make up an extremely heterogeneous group, arguably more so than any other age group (Grigsby, 1996). Yet, businesses often make facile generalizations by assuming older consumers only require leisure or medical products (Coughlin, 2017). In fact, one industry that has come under intense scrutiny is the anti-aging industry.

Attempts to control the process of aging date back to the early civilizations (Gruman, 2003; Olshansky et al., 2002). However, advances in science and medicine have led to a slew of interventions aimed at slowing, arresting, or reversing the process of growing older (Mehlman et al., 2004). This construction of aging as a medical problem was most notably articulated by Estes and Binney (1989) in what has since become a landmark study. They pointed out the influence of this biomedicalization of aging on the collective conscience, arguing that it gave rise to the perception of the aged body as a locus of disease and a site for repair.

Anti-aging messaging has famously permeated the beauty industry. Commercial and clinical enterprises offer a panoply of products, regimens, and treatments often touted as elixirs of youth (Fishman et al., 2008). Gerontologists have denounced the anti-aging industry as problematic in that it stigmatizes a natural transition, paints old age as the adversary, and ultimately incites a fear of growing older (Flatt et al., 2013). Some have also conceptualized the construct of anti-aging as an extension of patriarchal power (Healey, 1993) as well as a capitalistic framework designed to monetize feelings of insecurity (Williams, 2013).

At the same time, it is critical to highlight that the anti-aging industry has flourished precisely because of its ever-growing consumer base. The global anti-aging market was valued at approximately US$58.5 billion in 2020 and is anticipated to witness a compound annual growth rate of 7% between 2021 and 2026 (Statista Research Department, 2022). The reasons for partaking in anti-aging work vary from person to person, but in a society where beauty is tied to youth, it mostly constitutes a strategy for self-empowerment and for evading the marginalization that eventuates when one is visibly older (Clarke & Griffin, 2008; Negrin, 2002). Evidence suggests that individuals often undergo cosmetic procedures as a way to bolster confidence and improve self-esteem (Sadick, 2008). Going for minimally invasive, injectable treatments have likewise been found to improve psychological well-being and reduce appearance-related distress (McKeown, 2021).

To date, there has been a dearth of literature on the types of innovations designed for the older population. Accordingly, this study circles around the following questions: What are the latest innovations that have been designed for older adults? What are the objectives of these innovations? What messages of old age are being communicated by these patent grants? What do the patent grants reflect about inventors’ views on old age? We conduct a qualitative content analysis of patent grants to answer these questions.

## Method

### Data Set

We retrieved data from the U.S. Patent and Trademark Office using the Bulk Data Storage System (BDSS) Application Programming Interface (API) version 1.1.0. The API allows us to download patent and trademark data within the BDSS in bulk form and to search for data by date. We used the Patent Grant Full Text data set, which contains the concatenated full text—excluding images and drawings—of each patent grant document issued every Tuesday from January 6, 1976, to the present.

To identify the latest innovations targeted at the older population, we collected patents issued in 2021, specifically those issued from January 5, 2021 to December 28, 2021. Patents were compiled if they had a title or abstract containing the following search terms: aging, ageing, elder(s), elderly, old(er) adult(s), older(er) people, old(er) person(s), senior citizen(s), nursing home resident(s), retiree(s), young-old, youngest-old, middle-old, old-old, oldest-old, senesce, senescing, senescent, and senescence. Most of these terms were derived from earlier work on terms used to refer to older people (Ng, 2021b; Ng, Chow, et al., 2022; Ng et al., 2021a; Ng, Indran, et al., 2022b; Ng & Indran, 2022d, 2022e, 2023b; Ng & Lim-Soh, 2021). In the same search query, we excluded abstracts or titles with the following terms: baby, babies, child, children, teenager(s), adolescent(s), toddler(s), and kid(s). In total, 326 patents were collected. Upon removing irrelevant data, that is, patents not related to older adults (*N* = 206)—for instance, patents that looked at the aging of wine—the data set consisted of 120 patents.

### Content Coding

Similar to earlier research (Ng & Indran, 2022a, 2022b, 2022c, 2023c), the coding rubric was developed through both inductive and deductive modes of reasoning (Armat et al., 2018). In inductive content analyses, codes are derived directly from the data (Hsieh & Shannon, 2005). In contrast, analyses guided by a directed or deductive approach start with the identification of an initial set of codes based on prior literature (Hsieh & Shannon, 2005). We adopted both inductive and deductive approaches to ensure that certain fundamental assumptions informed the analysis while also aware that new categories would surface inductively (Armat et al., 2018).

To create a preliminary codebook, we first identified several categories based on past literature regarding innovations for older adults. The content analysis was subsequently conducted in a few stages, with each abstract read twice by two researchers trained in gerontology to ensure familiarity with and immersion in the data (Hsieh & Shannon, 2005). The goal of the first reading was to ascertain the validity of the initial set of categories, as well as to generate codes systematically across the whole data set. Each researcher modified the codebook independently until all variables were refined and clearly defined. During this first reading, a new category was added whenever an abstract featured a particular trait which could not be suitably coded into the existing categories, and which was recurrent in the data. During the second reading, the two coders had discussions where any discrepancies were reviewed and adjudicated to guarantee rigor in the content analysis. At this juncture, the two coders discussed the meaning of the codes, confirmed the relevance of the codes to the research question, and noted areas of major overlap to confirm the codebook.

The percentage agreement between the two raters was 95.5% with a weighted Cohen’s kappa of 0.92 (*p* < .001), indicating high interrater reliability. Three themes surfaced from the content analysis and the frequency of each theme was identified after the analysis. As mentioned in prior scholarship, categories in a content analysis need not be mutually exclusive although they should be internally homogeneous (i.e., coherent within themes) and externally heterogeneous (i.e., distinct from each other) as far as possible (Bengtsson, 2016).

## Results

### Summary of Insights From Content Analysis of Patents

**Figure 1.**
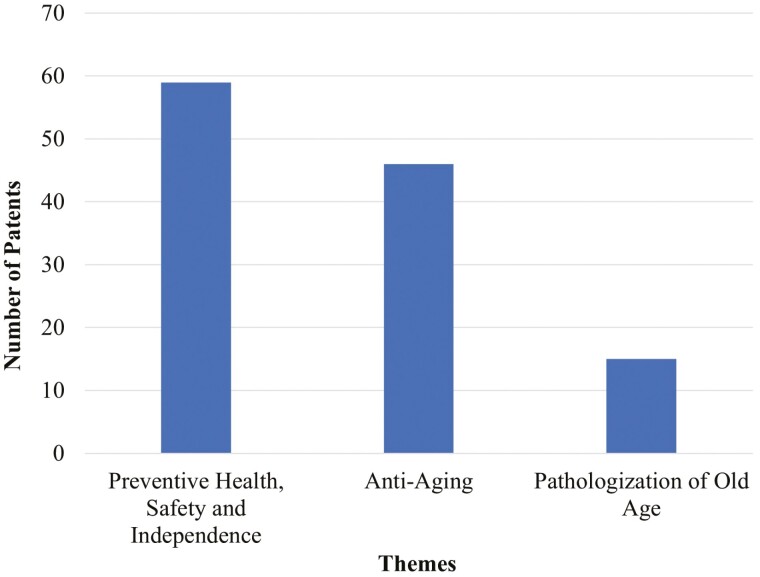
Themes of patents designed for older adults that were awarded in 2021.

Three themes emerged from our content analysis of 120 patent grants. About half (49.2%; *N* = 59) of the patents were about “Preventive Health, Safety, and Independence” (Theme 1). Patents in this category focused on making the environment more age-friendly, tackling age-related decline, or prolonging the independence of older adults. Theme 2 “Anti-Aging” appeared in over a third of the patents (38.3%; *N* = 46). Patents in this theme pertained to substances seeking to cure or reverse aging, as well as compositions for eliminating physical markers of old age such as wrinkles. The final theme “Pathologization of Old Age” (Theme 3) made up 12.5% of the patents (*N* = 15). Patents in this theme positioned old age as an illness. The themes are summarized in Figure 1. Examples of abstracts of patents can be found in Table 1.

**Table 1.** Examples of Abstracts of Patents Designed for Older Adults That Were Awarded in 2021

Theme	Abstract of sample patent
Preventive Health, Safety, and Independence (49.2%)	• A manually actuated drug-injecting device is configured such that the grip strength of the entire hand (i.e., majority of fingers closing toward the palm or heel of the hand) is employed to discharge medication through a hypodermic needle and into a patient’s body. The device is well suited for delivering medications with high viscosity and/or by patients (e.g., elderly patients) with reduced finger strength and dexterity.
	• A shower spray chair apparatus for assisted washing for elderly or disabled people includes a chair back and a chair bottom coupled to a chair frame.
	• The present invention consists of a multimedia fall prevention system serving the fall prevention needs of older adults.
	**•** The present invention relates to a new integrated, holistic approach to empower older adults to enhance their quality of life and independence through a personalized lifestyle and nutrition program.
Anti-Aging (38.3%)	• Disclosed herein are compounds for use in lightening skin, treating uneven skin pigmentation and/or improving the appearance of aging skin, as well as methods utilizing the compounds, and anti-aging compositions comprising the compounds and a pharmaceutically acceptable carrier.
	• A transgenic *Caenorhabditis elegans* model provided in the present invention is an animal model in which gln 5'-tsRNA is overexpressed such that aging is inhibited.
	• The invention relates to a composition, in particular to an oral anti-aging composition for treating hair aging.
	• Beneficial topical compositions for treating scar or aging skin are provided. The composition may also be used cosmeceutically in the treatment of aged skin, and may include phosphatidylserine, vitamins, and other beneficial anti-aging ingredients.
Pathologization of Old Age (12.5%)	**•** The present disclosure relates to a resveratrol delivery system having modified resveratrol composition wherein the active ingredient is stable colloidal resveratrol nanoparticles in unconjugated form. The said composition and its process thereof can be used in numerous applications for treatment of a mammal susceptible to or afflicted with insulin resistance, metabolic syndrome, aging, apoptosis, inflammation, stress resistance, cancer, [...].
	• The present invention relates to novel hydroxyl compounds, compositions comprising hydroxyl compounds, and methods useful for treating and preventing a variety of diseases and conditions such as, but not limited to aging, Alzheimer’s disease, cancer, cardiovascular disease, diabetic nephropathy, [...].
	• Inhibitors of ALCAT1 are described having the general formula: (i) these compounds offer a treatment for aging and age-related diseases.
	• Compositions and pharmaceutical compositions including a dendrimer-resveratrol complex and methods for making and using the compositions are described herein. Methods of treating cancer, cardiovascular disease, cardiac failure, diabetes, Alzheimer’s disease, Parkinson’s disease, [...] aging, [...] including administering a therapeutically effective amount of the compositions to a subject in need are also provided.

*Note*: ALCAT1 = Acyl-CoA:lysocardiolipin acyltransferase 1.

### Preventive Health, Safety, and Independence (Theme 1; 49.2%)

Inventions filed under this category focused on promoting the physiological health, safety, and independence of older adults. For instance, a particular composition was described as having the potential to “improve the quality of life of the elderly population” by “protect[ing] cells from oxidative stress.” Another invention found that the “enzyme inhibition” observed with certain “chemically distinct polyphenols” was associated with “healthy aging.” One invention sought to promote skin health by “assisting users with proper application of applied-to-skin sunscreen.” A “chewable eye health formulation” was designed in view of how the “risk of visual impairment[,] including age-related macular degeneration and cataracts[,] increase[s] with age.” There were many other inventions meant for “treating and/or preventing aging-related conditions” such as “age-related dementia” and “Parkinson’s disease.”

Several grants were about meeting specific age-related needs. A “manually actuated drug-injecting device” was created to help “patients (e.g., elderly patients) with reduced finger strength and dexterity” to deliver medications with high viscosity. To “account for the effects of the aging of the human eye,” a group of applicants came up with a gaming device that adjusts the brightness, vibrancy, and colors of displayed content using “age-determining data.”

Ensuring the safety of older adults was the objective of some inventions. One application drew attention to a statistical finding that “every year, approximately one in three older adults who are over 65 will fall.” The invention was a “multimedia fall prevention system.” Other innovations include a “composite safety toothbrush,” a “shower spray chair apparatus,” a “personal airbag device for preventing bodily injury,” a mechanism that would detect “vital signs” as well as a “resting device” for “improving [the] prediction and detection of adverse events.”

A handful of inventions looked at prolonging the independence of older adults. A “personalized lifestyle and nutrition program” was designed to “empower older adults to enhance their quality of life and independence.” There was an invention created for monitoring older adults in a smart home environment. Another invention was an apparatus “for closing a car door from the inside of a car using an elongated handle.” A portable heart monitor was developed to be used from one’s home to better “increase patient compliance.”

### Anti-Aging (Theme 2; 38.3%)

Patents that reflected anti-aging ideologies were clustered into this theme. Implicit in many of these applications is the idea that aging is undesirable. The bulk of these patents were about “treating cutaneous signs of aging.” A typical example of an invention would be “anti-aging compositions” meant for “preventing, slowing, and reversing skin aging.” Other verbs meant to cast skin aging as a defect—“inhibit,” “delay,” and “repair”—were commonplace.

“Fine lines” and “wrinkles” were insinuated as being flaws or abnormalities. One abstract proposed the use of “compounds, cosmetic or dermopharmaceutical compositions” for “treating, protecting, and/or improving the condition and/or aesthetic appearance of skin, for example, treating, preventing, ameliorating, reducing and/or eliminating fine lines and/or wrinkles of skin, or improving the appearance of fine lines and/or wrinkles of skin.” Compositions “comprising vitamin E, vitamin C and white tea extract” were proclaimed to “reduce signs of wrinkles and improve other skin conditions, such as increased elasticity and skin softness.” One invention was highlighted as containing “beneficial anti-aging ingredients,” intimating that to age would be an unwelcome consequence.

A few of the inventions were anti-aging substances. One of them pinpointed the “transgenic *Caenorhabditis elegans*” as an ideal model organism for investigating “anti-aging mechanisms.” The invention was said to be contributing to research for “developing new anti-aging drugs and screening for age-inducing materials.” Other patents were about “reversing” the “normal aging process.” For example, an “anti-inflammatory and anti-oxidative nutraceutical composition” supposedly yielded “unexpected benefits in promoting health, including synergistic effects in fighting cancer, slowing aging, and in some cases, reversing aging.” One patent discussed the use of an “oral anti-aging composition for treating hair aging.”

### Pathologization of Old Age (Theme 3; 12.5%)

Patents in this section positioned the aged body as a diseased entity. Medical patter like “symptoms” and “treat” surfaced in these abstracts, presenting the process of aging as an illness requiring cure. One invention advanced the use of combination therapies containing “disulfiram and one or more additional ingredients” as a method for “reducing a symptom of aging.” Another abstract mentioned that the inhibitors of Acyl-CoA:lysocardiolipin acyltransferase 1 “offer a treatment for aging.”

In some applications, old age was listed as one of many “diseases” to be managed. For instance, compositions comprising hydroxyl compounds were singled out as “useful for treating and preventing a variety of diseases and conditions such as, but not limited to aging, Alzheimer’s disease, cancer, cardiovascular disease, diabetic nephropathy, diabetic retinopathy, a disorder of glucose metabolism, dyslipidemia, dyslipoproteinemia, hypertension, impotence, inflammation, insulin resistance, lipid elimination in bile, obesity, oxysterol elimination in bile, pancreatitis, pancreatitius, Parkinson’s disease, a peroxisome proliferator-activated receptor-associated disorder, phospholipid elimination in bile, renal disease, septicemia, metabolic syndrome disorders (e.g., syndrome x), thrombotic disorder.”

In another patent, a type of resveratrol composition—with “stable colloidal resveratrol nanoparticles in unconjugated form” as the “active ingredient”—was marketed as being able to treat those “afflicted with insulin resistance, metabolic syndrome, aging, apoptosis, inflammation, stress resistance, cancer, cardiovascular disease, muscular dystrophy, low fertility rates, or any combination thereof.”

## Discussion

The rise in proportion of the older population has prompted calls for more age-friendly innovations (Ng, Lim, et al., 2020; Sweetland et al., 2017). This is the first study to analyze a data set of patent grants related to inventions for older people. We performed a content analysis of these grants to understand the types of messages being conveyed about old age in the innovation ecosystem. Three main types of patents emerged from the analysis. Half of them focused on “Preventive Health, Safety, and Independence” (Theme 1). Over a third of them were about “Anti-Aging” (Theme 2). The remaining inventions revealed a “Pathologization of Old Age” (Theme 3).

Longer life spans do not necessarily equate to longer health spans—the period in which one is free from serious disease (Hansen & Kennedy, 2016). Solutions that assist in tackling age-related decline are therefore critical for an aging society. About half of the patented publications analyzed were innovations geared toward enhancing older adults’ physical and cognitive health, making the environment more age-friendly, or prolonging their independence (Theme 1).

More than a third of the patents embraced anti-aging ideals (Theme 2), with the majority in the form of beauty products focused on eliminating visual signifiers of old age such as wrinkles and fine lines. The creation of such products reflects a continued portrayal of old age as a process that requires physical intervention. Theme 2 also contained patents that promised to delay or reverse aging. Gerontologists have taken issue with the scientific quest for immortality in the medical field, maintaining that interventions seeking to cure or reverse aging merely portray old age as the enemy (Flatt et al., 2013). They assert that the ultimate goal ought to be a prolongation of health rather than of youth (Flatt et al., 2013). Additionally, some have highlighted that anti-aging medicine invokes a series of ethical implications, such as the likelihood that only the well-off will be able to afford such treatments, hence exacerbating existing inequalities (Partridge et al., 2009). Anti-aging drugs have also been critiqued in the way that they run contrary to natural laws and devalue the worth of human life (Partridge et al., 2009).

Although aging is a normal, biological process, over 10% of the patents pathologized it as an ailment in need of curing (Theme 3). This biomedicalization of aging has conditioned society to view aging as abnormal (Estes & Binney, 1989; Ng et al., 2015; Ng & Chow, 2021). Such negative views of old age may be extended to those who are aging. When assimilated from one’s culture, age stereotypes eventually become self-definitions that affect health in later life. Older adults who endorse negative age stereotypes tend to have a lower sense of efficacy, higher risk of depression, and poorer cardiovascular health. Meanwhile, those who hold positive age stereotypes typically have better well-being, improved functional health, and greater longevity (Levy, 2009; Levy et al., 2000, 2002).

### Recommendations

Our results reveal the need to expedite processes to improve the experience of aging, particularly given the reputation of the United States as a cradle for innovation (Feulner, 2018). We provide several recommendations.

First, rather than combing for ways to cure or reverse what is only a natural and biological universal, effort could be concentrated on allowing older adults to better cope with age-related functional decline so that they can lead socially and economically active lives for a longer period. For example, incorporating the latest technologies into hearing aids or mobility devices may ease the transition of older adults into later life. With the right innovations, older adults can still lead healthy and independent lives while experiencing some of the natural changes in areas such as vision, hearing, and mobility (Sweetland et al., 2017).

Second, it is unclear whether anti-aging products are developed due to unconscious biases held by innovators working across various entities such as universities, research institutes, and commercial enterprises. Regardless, the success of the anti-aging industry speaks to the desire of many—young and old alike—to either maintain or reinstate their youthful image. This may be to the consternation of gerontologists and activists. Nonetheless, it is vital to ensure that older persons who elect to partake in anti-aging work are not shamed or marginalized for doing so.

Third, technology could be leveraged to improve the social connectedness among older people, prepare them for retirement, and stimulate lifelong learning. A robot powered by artificial intelligence was recently created to alleviate the effects of social isolation experienced by older persons (Schroeder, 2022). Certain social media applications, though not created specifically for older consumers, have also conferred upon them social (Baecker et al., 2014; Ng & Indran, 2023a) and cognitive (Quinn, 2018) benefits.

Fourth, governments should set goals to incentivize innovators to improve the quality of care dispensed to older adults. A case in point would be Singapore’s National Innovation Challenge on Active and Confident Ageing, which was launched to catalyze innovation and research that can transform the aging experience in the country (National Medical Research Council, 2022). As part of this challenge, the Singapore University of Technology and Design developed an application that improves the cognitive functioning of older persons by engaging them in various dual-language cognitive tasks. This application has been well received by older Singaporeans and has been effective in enhancing their verbal memory (Heng, 2022).

Fifth, there is a need for innovators at universities, research institutes, and commercial enterprises to actively involve older adults in design processes particularly given the heterogeneity of this demographic. Interviews and focus group discussions could be held to ensure their diverse needs are taken into consideration. Companies should also be informed that they can gain a competitive advantage if they have products catered to the needs and demands of a group with burgeoning spending power (Kluge et al., 2014).

Finally, to foster healthy aging, innovators must understand that growing older is a multifaceted experience that covers biological, cognitive, physical, psychological, and social aspects (Halaweh et al., 2018; Phelan et al., 2004). To this end, gerontologists and more broadly social scientists could be included in the development of new products. Such collaborations will better attune innovators and designers to the complex issues to be considered in fulfilling the needs of older persons.

### Limitations and Future Directions

This study has two key limitations. First, our study only focused on innovations in the form of patents that do not necessarily include products created by profit-driven commercial entities in response to consumer demand. Second, the data set analyzed represents a subset of all inventions created to serve older consumers. Specifically, it comprises only a year’s worth of patents. Furthermore, not every invention will be patented for several reasons. For example, there are inventions that are simply not patentable (Fontana et al., 2013). The cost of filing a patent application might also be prohibitive for some. Moreover, there may be innovators who are not keen on holding an exclusive right to a particular invention. In addition, there could be innovations underlying successful products that inventors or companies are not willing to make public. Our data set is therefore not indicative of the entire field of innovation for older adults.

Directions for future research are aplenty. First, as this study only queried age-related terms, future studies could explore alternative search terms to corral the relevant data. Other methodologies could also be employed to understand the state of innovation for the older population. Second, it would be worthwhile to learn how innovations for older adults have changed over time particularly as this segment has been growing the fastest. Third, as citations are a measure of how valuable patents are, there is scope for an analysis on what messages about old age are being conveyed in the most cited patents. Fourth, interviews, surveys (El-Muttardi et al., 2005; Ng, Allore, et al., 2020; Ng et al., 2016; Ng & Levy, 2018; Ng & Rayner, 2010) and big data analytics (Giest & Ng, 2018; Ng et al., 2023; Ng, Indran, et al., 2022a; Ng & Tan, 2021a) could be employed to understand the types of beliefs that drive various inventions to see if innovators themselves harbor age stereotypes that they may not be aware of. Fifth, studies could explore inventions in other countries that are well-known for innovation such as Switzerland and Sweden (Soumitra et al., 2021). Understanding how inventions differ across cultures (Ng, 2021a; Ng et al., 2021b; Ng & Indran, 2021a, 2021b; Ng & Tan, 2021b, 2022) may also present a fruitful avenue for future analysis.

## Conclusion

An aging population creates fertile ground for devising innovations for older adults. In this study, we evaluated the state of innovations for an aging population. While there are indeed innovations aimed at optimizing the well-being of older adults, there are also inventions that see old age as a problem to solve. As the world experiences a demographic shift, it is imperative that collective ingenuity be harnessed to build an ecosystem conducive to all facets of the aging experience. Through the judicious application of innovation, old age can be embraced and celebrated without the impediments of ageist notions.

## Data Availability

Data are publicly available at https://www.uspto.gov. This study was not preregistered.
